# Serotonin biosynthesis emerges as a metabolic driver in prostate cancer therapy resistance

**DOI:** 10.1172/JCI210677

**Published:** 2026-11-16

**Authors:** Kevin Holm, Neil A. Bhowmick

**Affiliations:** Department of Medicine, Cedars-Sinai Medical Center, Los Angeles, California, USA.

## Abstract

Advanced prostate cancer frequently escapes androgen receptor pathway inhibition through mechanisms including restored androgen signaling, lineage plasticity, and metabolic adaptation. Neuroendocrine prostate cancer (NEPC) can emerge as an androgen-insensitive lineage and has mostly been studied in the context of its transcriptional and epigenetic reprogramming. However, evidence increasingly supports the requirement for metabolic rewiring across a variety of pathways in the transition to NEPC. In this issue of the *JCI*, Wei et al. demonstrated that serotonin metabolism regulated by tryptophan hydroxylase 1 (TPH1) stabilizes the plastic state of NEPC by supporting serotonin-based posttranslational modification (serotonylation) of mTOR and FOXM1/E2F1 activation. These findings position TPH1 as both a neuroendocrine marker and a functional mediator of hormone therapy resistance. Because metabolic escape can involve many pathways, biomarker-guided patient selection will be required for translating these findings to an intervention. Accordingly, targeting TPH1 may delay or reverse therapy-induced NEPC in selected patients.

## Introduction

Advanced prostate cancer is effectively suppressed by androgen receptor (AR) inhibitors due to its dependence on androgen signaling. Yet tumor adaptation limits the durability of this strategy. Some tumors engage diverse mechanisms to restore androgen signaling in the context of targeted antagonists, including AR amplification, mutation, splice variants, enhancer rewiring, or intratumoral steroid synthesis (1, 2). Others become less dependent on AR signaling and acquire basal, stem-like, or neuroendocrine (NE) features (3–5). Neuroendocrine prostate cancer (NEPC) is a subtype of castration-resistant prostate cancer that arises in response to AR pathway inhibitor (ARPI) treatment as an AR-indifferent, epigenetically and transcriptionally reprogrammed state (4, 5). The role of cytokines in paracrine mechanisms of prostate tumor adaptation has been a focus of therapy resistance studies (6–8). Emerging data suggest that castration-resistant prostate cancer is metabolically distinct from androgen-sensitive prostate cancer (9–11), highlighting the potential to identify and target meaningful metabolic contributions to resistance.

## Metabolism as a driver of hormone therapy escape

Several metabolic pathways have been associated with resistance to hormone therapy. Fatty acid oxidation programs, including acyl-CoA synthetases, regulate ferroptosis resistance and sensitivity to ARPI in prostate cancer cells (12). Peroxisomal oxidative stress induced by AR inhibition can promote ARPI resistance through PPARα-dependent pathways (13). Steroid metabolism, including adrenal androgen utilization that is dependent on the extragonadal androgen synthesis enzyme HSD3B1, contributes to ARPI treatment response (14, 15). Glucose transporters, solute carriers, bile acid metabolism, and one-carbon metabolism have also been linked to ARPI resistance and progression to NE phenotypes (16). Among these metabolic contributors to therapeutic resistance, tryptophan hydroxylase 1 (TPH1) adds a conceptually distinct mechanism by modifying the amino acid metabolism underlying monoamine synthesis that supports lineage plasticity. TPH1, the rate-limiting enzyme in serotonin synthesis outside the brain, supports serotonin availability for protein posttranslational modification of proteins (e.g., histone serotonylation), driving prostate cancer lineage plasticity. Serotonin can further rewire glycolysis, mitochondrial activity, immune metabolism, and therapy resistance in NEPC (17).

Wei and colleagues report in this issue of the *JCI* that TPH1 was increased in therapy-induced models of NEPC, genetically driven NEPC models, and patient-derived xenograft systems (18). They further showed TPH1 depletion suppressed proliferation, invasion, sphere formation, NE marker expression, organoid growth, and xenograft growth in NEPC models. The key mechanistic advance is the proposed intracellular serotonin/mTOR axis (18): rather than acting primarily through extracellular serotonin receptors, TPH1-derived intracellular serotonin supported transglutaminase-dependent serotonylation of mTOR at Gln2453, enabling mTOR activation and downstream induction of FOXM1 and E2F1 programs.

These findings are interesting because they connect serotonin, which is canonically associated with NEPC (19), to a growth and lineage-control pathway. They also provide a posttranslational mechanism by which metabolism can rapidly alter signaling topology. Thus, TPH1 can serve as a NE marker as well as a metabolic mediator that helps stabilize the plastic NE state. This builds on past work demonstrating glucocorticoid receptor signaling and glutamine metabolism as complementary drivers of mTOR-mediated NEPC progression, supporting lineage plasticity, metabolic adaptation, and resistance to AR-targeted therapies (10, 20). These pathways contribute to androgen receptor pathway modulator resistance (APMR), a major clinical challenge in patients undergoing treatment for prostate cancer.

## Therapeutic implications of serotonylation

By linking tryptophan metabolism to intracellular serotonin production, mTOR serotonylation, and FOXM1/E2F1 activation, Wei and colleagues place serotonin metabolism within the broader circuitry of prostate cancer lineage plasticity. This opens an emerging area of investigation in which amino acid metabolism can stabilize AR-low and NE states. Tryptophan metabolism adds to the already long list of mechanisms of APMR, including lipid metabolism, ferroptosis defense, peroxisomal oxidative stress, steroid metabolism, glucose transport, and one-carbon metabolism (9, 21). Accordingly, it will be valuable to identify biomarkers to help prioritize interventional strategies.

One of the key advances Wei and colleagues uncovered is that elevated mTOR signaling releases NEPC cells from metabolic constraints of the microenvironment (18). Previous studies have highlighted that the profound metabolic changes supporting the shift from adenocarcinoma to NEPC occur alongside serotonin secretion from NEPC cells (22). With mTOR inhibition having limited efficacy in prostate cancer treatment, Wei and colleagues identified transglutaminase 2 (TGM2) as a critical component in the binding of serotonin to mTOR (18). They treated prostate cancer cells with a competitive inhibitor for TGM2, monodansylcadaverine (MDC), and observed reduced activation of mTOR and consequent NE-like phenotype (18). Interestingly, MDC is a recognized autophagy marker, and autophagy is an implicated mechanism for NE differentiation.

Most notable, however, was the authors’ use of an existing TPH1 inhibitor, LX1606 (telotristat ethyl), a compound with limited blood-brain barrier penetration. As might be expected, TPH1-high prostate cancer cells were more sensitive to LX1606, with up to 80% lower IC_50_ compared with TPH1-low cells. Consequently, TPH1 enzymatic activity in TPH1-high cells was significantly reduced by LX1606 treatment, resulting in a decreased serotonylation rate (18). Furthermore, LX1606 sensitized enzalutamide-resistant C4-2B human prostate cancer cells to enzalutamide with increases in AR and prostate-specific antigen (PSA) protein expression. These results were validated in several xenograft mouse models, resulting in a substantial reduction of tumor volume, serotonin levels, and NE marker expression (18). There was also no significant change in body weight or in liver or kidney pathology in the animal models, supporting its potential clinical viability.

As LX1606 is approved in adults for carcinoid syndrome diarrhea inadequately controlled by somatostatin analog therapy, the current findings provide a direct translational bridge from mechanism to intervention. But repurposing LX1606 as an intervention in prostate cancer would require prospective biopsies or validated liquid/tissue biomarkers, since PSA alone is poorly suited to detect lineage conversion to NEPC. Similarly, blood serotonin levels would not be a reliable biomarker. Rather, 24-hour urinary 5-hydroxyindoleacetic acid, the principal serotonin metabolite, in conjunction with biopsy confirmation of NEPC could support a trial to test LX1606 efficacy in combination with an ARPI.

## Current understanding and remaining questions

The study by Wei and colleagues extends a growing body of work showing that resistance to ARPI is a result of a spectrum of adaptive states that include restored AR signaling, nuclear receptor bypass, stromal support, altered nutrient use, and lineage plasticity (Figure 1). Foundational studies from Beltran and colleagues established treatment-emergent NEPC as an AR-indifferent state that diverges through epigenetic and transcriptional reprogramming yet retains shared genomic ancestry (4, 5). As one example of metabolic reprogramming contributing to NEPC differentiation, glucocorticoid receptor–mediated ARPI bypass can itself be shaped by alterations in steroid metabolism (23). Glutamine metabolism similarly contributes to castration resistance through tumor-intrinsic mechanisms, and stromal mTOR supports NE differentiation and ARPI resistance (10, 24). Lipid metabolism, ferroptosis defense, glucose transport, bile acid signaling, and one-carbon metabolism add further layers to this adaptive landscape (25, 26). Prior work has shown that NEPC can synthesize, accumulate, transport, and secrete serotonin (19). The current study places serotonin as a metabolic input capable of stabilizing the resistant lineage state that results in ARPI resistance with low PSA, loss of luminal identity, and acquisition of NE features (18). The remaining challenge is how to identify the dominant metabolic dependency in a given tumor at the clinically actionable moment.

This work also identifies the TPH1 inhibitor LX1606 as a potential sensitizer to ARPI-resistant prostate cancer by inhibiting the shift to a NE phenotype. However, it is important to consider that TPH1 is a serotonin biosynthesis enzyme. While the authors provide a strong mechanistic claim that the serotonylation of mTOR is a key component of this process, it is likely that there are more potential targets of TPH1-dependent serotonylation that might be relevant, especially histone proteins. Further work mapping the full “serotonome” might reveal a broad range of viable therapeutic targets for inhibiting ARPI-driven NEPC, especially in its convergence with epigenetic regulation or metabolic shift. The mTOR serotonylation finding is compelling, but serotonin is unlikely to modify only one substrate. Near-term solutions could include mass spectrometric profiling of antiserotonylation antibody pull-down during adenocarcinoma-to-NEPC transition, with appropriate controls to limit nonspecific signals. Other studies of NEPC have implicated secreted serotonin in neutrophil remodeling and liver metastasis involving histone serotonylation that may contribute to lineage plasticity, metabolic state, immune suppression, or metastatic colonization (17). These mechanisms are not mutually exclusive.

The broader implication is that resistance to hormone therapy should be viewed less as a binary question (AR-on versus AR-off) and more as an adaptive state selection problem. The divergence in response between TPH1-high and TPH1-low cells also points to the need for further investigation into the tumor microenvironment and the cell populations that emerge. If TPH1 activity is required early during lineage transition, then inhibiting this enzyme may delay or prevent NEPC emergence. If TPH1 is required after NEPC is established, then its inhibition may be combined with ARPI or other approaches. The work by Wei and colleagues is timely because recent studies increasingly frame hormone therapy sensitivity in prostate cancer not only as a problem of AR reactivation or lineage transcription factors but also as a problem of metabolic state. The inclusion of serotonin biosynthesis and intracellular serotonylation within the circuitry of lineage plasticity shifts the conversation away from doubling down on ARPI and highlights metabolic wiring involved in the response to hormone therapy. The next step is to determine which patients are truly dependent on this serotonin-centered axis, when that dependence emerges, and how best to combine axis inhibition with therapies that target the parallel metabolic and epigenetic routes of hormone therapy escape.

## Manuscript information

### Author contributions.

KH contributed to writing, reviewing, and editing the manuscript. NAB conceptualized the commentary and contributed to writing, reviewing, and editing the manuscript.

### AI disclosure.

The authors declare that ChatGPT was used to identify past publications in the field.

### Conflict of interest.

The authors have declared that no conflict of interest exists.

### Funding.

This study was supported by the following:

- A grant from the NIH National Cancer Institute (R01CA292654) to NAB.

This work is the result of NIH funding, in whole or in part, and is subject to the NIH Public Access Policy. Through acceptance of this federal funding, the NIH has been given a right to make the work publicly available in PubMed Central.

## Figures and Tables

**Figure 1:**
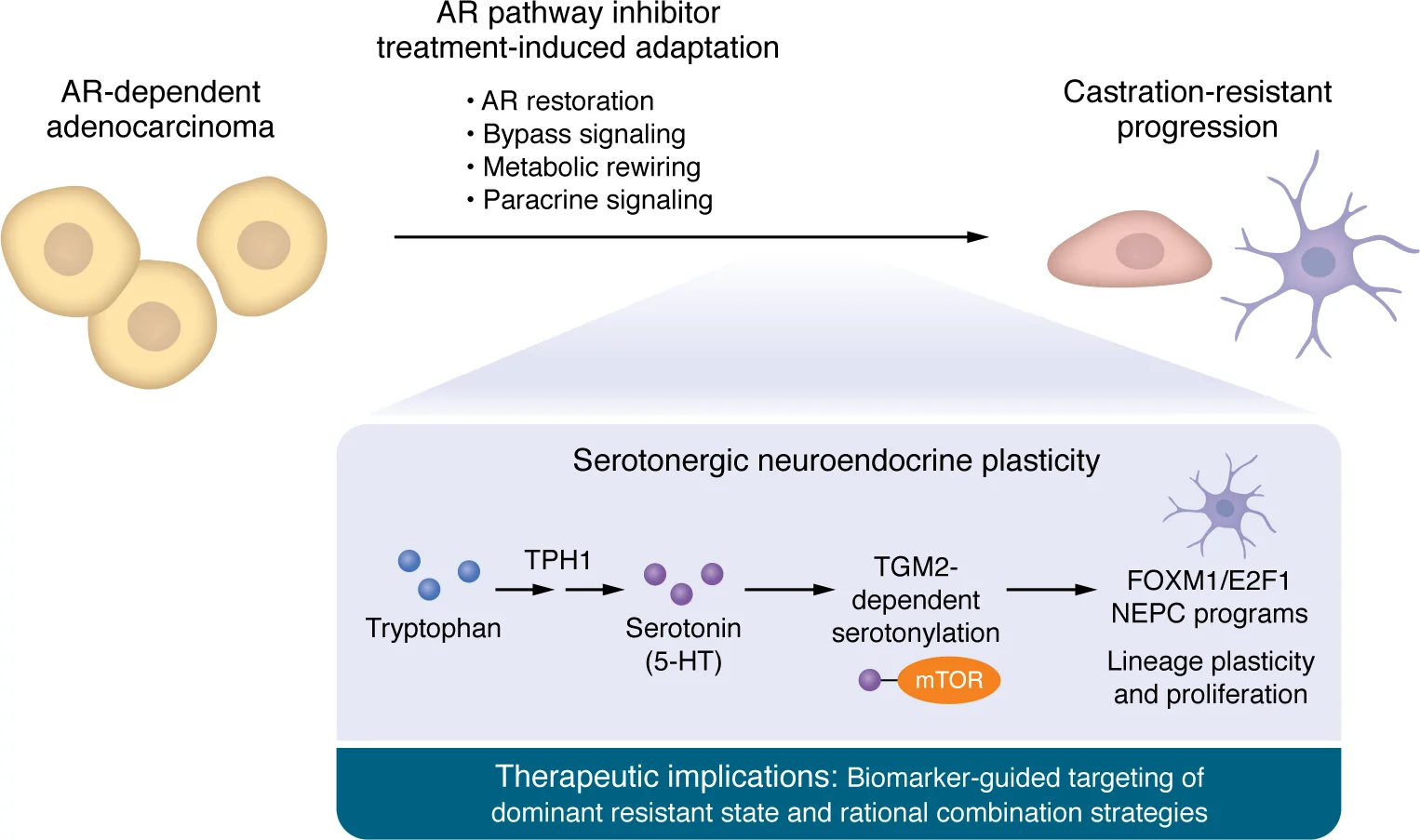
Drivers of hormone therapy escape. Prostate adenocarcinoma under androgen receptor (AR) pathway inhibition induces multiple treatment-resistant states. The heterogenous resulting tumor cells can develop resistance through AR signaling restoration, bypassing AR signaling through glucocorticoid receptor activation, metabolic rewiring, and transformation of the paracrine signaling axis. Wei et al. (18) connected serotonin metabolism to neuroendocrine subtypes of treatment resistance, showing that tryptophan hydroxylase 1 (TPH1) upregulation in models of neuroendocrine prostate cancer (NEPC) increased tryptophan conversion to serotonin, supporting serotonylation of targets such as mTOR that contribute to a neuroendocrine differentiation state.

## Version 1 11/16/2026

Electronic publication
